# Rural pipeline and willingness to work in rural areas: Mixed method study on students in midwifery and obstetric nursing in Mali

**DOI:** 10.1371/journal.pone.0222266

**Published:** 2019-09-09

**Authors:** Cheick Sidya Sidibé, Ousmane Touré, Jacqueline E. W. Broerse, Marjolein Dieleman

**Affiliations:** 1 Institut National de Formation en Sciences de la Santé, Bamako, Mali; 2 Athena Institute for Research on Innovation and Communication in Health and Life Sciences, Vrije Universiteit, Amsterdam, Netherlands; Liverpool School of Tropical Medicine, UNITED KINGDOM

## Abstract

The availability and retention of healthcare professionals in rural areas and remote areas is a challenge to the health sector worldwide. Attracting people who are most likely to be interested in rural practice can be conducive to the sustainable availability of health services in rural areas where health facilities are typically understaffed and existing professionals often underqualified. This article aims to contribute to the rural pipeline evidence, and reports on the findings of a study that investigated the career choices of midwifery and obstetric nurse students in Mali. The article enquires into the relationship between their intention to practice in rural areas and the different components of the rural pipeline. A mixed method study using a survey, semi-structured interviews, and document analysis was conducted from October to December 2017 on third-year midwifery and obstetric nurse students and school-managers. Descriptive statistics and bivariate analysis were performed for quantitative data, and content analysis was carried out for the qualitative data. Key findings suggest that students prefer urban locations for living and for work. The intention to work in rural areas seems to be influenced by the current living situation (currently living in a rural area) or having attended secondary school or professional training in rural areas.

## Introduction

Qualified and available health care providers are essential for delivering quality health care and improving the health status of populations. However, there is an acute shortage of qualified professionals in low-income countries that bear the greatest burden of maternal and child illnesses and deaths. According to its latest Demographic and Health Surveys (DHS IV and V), maternal mortality in Mali has declined from 464 to 368 deaths per 100,000 births [1]. But it is still among the highest in the world, far beyond the 70 per 100,000 live births targeted by the Sustainable Development Goals by 2030 [2]. Among factors that contribute to this mortality ratio is the lack of effective health services in rural locations [3,4], and limited access to healthcare services, resulting from a lack and maldistribution of human resources for health. To ensure universal health coverage and to make progress towards the reduction of maternal mortality, achieving equitable distribution of health workers between urban and rural areas is essential. This is particularly crucial for midwives and obstetric nurses, two front-line health professionals in Mali who are qualified to provide health services for pregnant women and newborns. However, the distribution of midwives and obstetric nurses across the health system is very imbalanced. As of 2017 there were an estimated 1,529 midwives in the country with one midwife for 2,775 inhabitants in Bamako, one for 28,348 in Kayes and one for 38,050 in Mopti [4].

Attracting people who are most likely to be interested in rural practice can be conducive to sustainable availability of health services in undeserved and rural areas [5]. Health professionals with a rural background are believed to be more likely to practice in rural locations [6,7]. Attracting students with a rural background to health studies or providing rural exposure to students during their training in an attempt to attract them to rural practice is referred to as rural pipeline [8]. The concept of rural pipeline has four components [9]: i) advocating health professions among rural students, ii) ensuring that more rural students are selected into programs, iii) developing a curriculum oriented towards rural health and rural exposure during training and iv) ensuring retention of health workers in rural areas through educational and professional support. Originally used for medical students, the approach is now applied to all health workers, including midwives and nurses [10].

Various studies and reviews in high- and middle-income countries have contributed to the evidence base of the effects of implementing (parts of) the rural pipeline. Literature reviews on rural school programs attest to the positive impact of rural exposure on increasing attraction of students to rural practice in different settings. Johnson et al. in a 2018 review indicated that rural clinical placement programs and rural clinical schools have a positive association with rural practice locations when they are well designed and financially supported [11]. O’Sullivan et al. in a recent review reported that Australia’s immersion programs are associated with an increased rural supply of doctors at their career debut [12]. A review by MacQueen et al. states that growing up in a rural community is a predictor of choosing a rural practice location [13]. Another review found that medical students attending a rural campus or spending time in a rural area are more likely to practice in non-urban areas after graduation. The longer students engage with rural areas during their training, the more likely they are to work in such areas after graduation [14]. Rabinowitz showed that rural medical school training programs in the United States have increased the number of rural physicians and that their large-scale replication could have a major impact on access to health care for rural communities [15]. Wenghofer et al. [16] studied the rural educational experience of the Northern Ontario School of Medicine and found that 25,4% of their graduates were practicing in rural areas compared to 10,3% for students from other Canadians medical schools. Australia has developed a national program to enhance its rural medical workforce by recruiting students with a rural background and establishing rural clinical schools. Evaluating the regional result of the program, Kwan et al. showed that predictors of long-term rural practice were a rural background, rural clinic schools and bonded scholarships [17]. McGrail et al. explored in Australia the association between the region of practice and the region of rural training, combined with the region of secondary schooling and duration of rural training and the return rate of medical graduates in their rural training region in early career was related to their connection with the region through their medical training and /or their secondary schooling [18]. In United States, the University of Missouri School of Medicine has developed a Rural Track Pipeline Program with a preadmission program for rural students, a Community Program, Rural Track Clerkship and a Rural Track Elective Program for students. Quinn et al. assessed the effect of the program on the residency choice and practice location of students and found that over 57% of students who participated in the rural track clerkship program chose a rural location for their first practice [19]. In Europe, Carson et al. [20] have examined the relationship between rural origin and exposure and the preference for rural jobs in six European countries, finding that both rural origin and rural exposure contribute to the retention in rural areas; but not to the same extent in all contexts. For instance, the rural pipeline is less important for retention of practitioners in rural areas that are very close to cities. In South Africa, Stellenbosch University has successfully implemented rural pipeline in an attempt to strengthen medical education in rural and constrained areas. The rural education intervention has led to change in the attitudes of students towards rural practice. They feel more confident in their preparedness to be a doctor in rural areas [21–23]. In addition, a study by de Vries et al. showed that rural-origin graduates in South Africa are more likely to practice in rural areas than those from urban areas [24].

As in high- and middle-income countries, in low-income countries where health workers shortages in rural and remote areas hamper access to care, studies have shown a positive correlation between rural origin, rural exposure and the students’ likelihood of opting for rural practice [25–30]. In a survey among medical and nursing students from nine African and Asia countries, Silvestri et al. found that a long duration in a rural setting was likely to anticipate selecting a rural practice [25]. In a number of African countries, the same trend is observed. In Rwanda and Ethiopia, students who have grown up in a rural area are more willing to work in a rural area [26]. In the Ghanaian context, medical students with rural experience are shown to be likely to work in a deprived area after graduation [27]. In a pilot program at the University of Nairobi, a decentralized training program demonstrated the positive effects of increasing students’ ability and willingness to work in rural and underserved areas [28]. Uganda’s experience of community-based education for undergraduate health professions students’ resulted in an increased proportion of students who expressed intentions to practice in rural areas [29]. In Niger, a study on the motivations of obstetric healthcare personnel to work in rural areas suggests that rural origin may be a motivator for health workers to accept rural posting [30].

In Mali too, in an attempt to improve accessibility of care particularly in rural areas, initiatives such as the decentralization of training schools in rural regions have been taken during the last decades. According to human resources and schools’ managers, the development of public or private schools in regions has likely contributed to the increase of human resources for health in these areas. For instance, this seems the case of the Gao School of Health (EIG), that revised curricula to include rural health topics and of which 92% of graduates (N = 35 midwives) remained in the region. Other initiatives aimed at retaining staff in rural areas through training have been experimented with. An orientation course on rural practice for newly established rural doctors from 2003 to 2005 contributed to a positive impact on their preparedness for rural practice and increased retention in rural areas, with 50% still in rural practice after 4 years [31].

Overall, evidence shows that rural students’ selection and rural exposure during education has proven to be effective in different contexts. One can hypothesize that such initiatives might be effective in Malian context as well because of the similarities in the behavior of health workers. However, there is yet insufficient evidence whether or not rural pipeline components can be used to develop strategies to attract maternal health work force to rural and remote areas in Mali. This article aims to contribute to the rural pipeline evidence, and reports on the findings of a study that investigated the career choices of midwifery and obstetric nurse students in the Malian context. It looks into the relationship between their intention to practice in rural areas and the different components of the rural pipeline. It specifically focuses on the components of the rural pipeline applied in the Malian context for midwives and obstetric nurses to attract them to rural practice and asks whether there is a relation between the rural pipeline components in place and the intention to practice in rural areas. In this study, we define rural areas as areas located outside main cities and the Bamako region.

## Materials and methods

This study was a mixed method study and used a survey, semi-structured interviews and document analysis. Data were collected from October to December 2017.

### Study population and sampling

Midwifery and obstetrics nursing students who were enrolled in degree program in health sciences training schools constituted the study population of this study. Schools were selected using a maximum variation sampling strategy based on their attendance rate and their location, so as to include midwifery and obstetric nursing students from different regions and different sectors.

#### Selection of training schools

The study included six private schools, one (the only) public training school in Mali, and three of its annexes. The public training school, the National Institute for Training in Health Sciences (INFSS), has a central structure in Bamako and four regional schools. It is in charge of research and training of allied health workers in Mali. It provides training at Bachelor (license) and Master levels. Public schools in the regions are annexes of the INFSS and have no autonomy of management and operation. They provide training up to the bachelor level. Three of them have been included in the study. The private schools train midwives and obstetrics nurses. Of the 10 schools involved in the studies, only one was situated in a rural area; two were situated in Bamako and seven in other urban areas. There were no midwife training school located in rural areas. Table 1 gives the overview of training schools involved in the study.

**Table 1:** Overview of sampled training school.

	Public schools	Private schools
**School location**		
• Capital city (Bamako)	1	1
• Other urban areas	3	4
• Rural areas		1
**Type of allied health students trained**		
• Midwives	4	5
• Obstetric nurses	0	6
	**Graduated students per year**	
	**Midwives**	**Obstetric nurses**
**Schools capacity**		
INFSS	75	0
• Infss Bamako	30	0
• Infss Sikasso	15	0
• Infss Ségou	15	0
• Infss Mopti	15	0
Santé plus Bamako	20	40
Yolande Brestown Mopti	10	30
CSK Koro	0	10
Cftss Ségou	10	20
CSK Kayes	5	20
ETS Sikasso	10	25

#### Selection of respondents

For the quantitative component, all midwifery and obstetric nursing students in their last training year at the selected schools were invited to participate. They were contacted in the classroom by researchers at the end of a lecture with request to participate. Those who agreed to participate were included. We conducted a convenience sampling among final year students. Potential participants were asked if they were willing to participate and were interviewed after they had provided consent. School managers were included as key informants. All school managers from selected schools were asked to participate and all of them agreed to. In addition, school managers from a private school, Gao nursing school, in the northern region in Mali (Gao) were included because it is the first and was for many years the only school located in the northern region in Mali. The managers were telephoned with request for interview. After agreeing and giving consent they were interviewed.

### Data collection methods and process

For the quantitative component, data collection was done through a survey using a self-administered questionnaire. Questions were asked about personal characteristics, motives to join midwifery or obstetric nursing training, and job preference. The questionnaire was developed and pre-tested and slightly adapted.

For the qualitative component, semi-structured interviews were conducted in a private location with only the participant and the interviewer. For the students, themes explored were career choices, job search strategies, mobility, career aspiration and job preference. For the managers, questions were asked about the organization of the curricula, the availability of support for graduates in finding a job, training policies, and admission and accessibility policies, practices and criteria. Interviews with managers from Kayes, Sikasso, Ségou, Mopti and Gao were done through telephone calls. Interview guides were pre-tested with school managers in Bamako. All interviews were digitally recorded. Each interview lasted about 45 minutes. The interviews were conducted by CSS (Male, MD, PhD student), a male interviewer (MD) and female sociologist (MSc). All the members of the team were trained in qualitative interviewing and were bilingual (French and Bambara). The saturation of the data was obtained during the interviews.

Documents related to training policy and regulation were collected from selected schools when available and analyzed.

### Data processing and analysis

Quantitative data were analyzed with IBM SPSS Statistics version 24 using descriptive statistics to describe characteristics of participants. The Chi square test and the Fisher exact test were used for bivariate analysis of students’ characteristics and their intention to work in rural areas. The P value below 0.05 was considered statistically significant.

Following transcription, interview data were transcribed and analyzed using Atlas.ti qualitative software version 8.1.0 (31). A coding framework was developed according to the research objectives and themes, the transcripts were coded and analyzed, comparing and contrasting answers between different groups of respondents, different sources of data collection, and between respondents’ answers and documents.

### Ethics approval and consent to participate

Ethical approval for this study was sought and obtained from the Ethics Committee of the National Institute of Research in Public Health (N°23/2017/CE-INRSP). Participation was voluntary. Participants were able to leave the study any time they wanted. Written informed consent to participate was obtained from each study participant except for those interviewed by phone. In these cases we obtained verbal consent.

## Results

Result are presented about midwives and combine the perspectives of all participants groups. Data on obstetric nurses are shown when differences are found in comparison to the answers from midwives. We first present rural pipeline components in use, and then students’ perceptions about and willingness to practice in rural areas.

### Rural pipeline components in training schools

#### Strategies to attract students

The training of midwives and obstetric nurses is provided by the public schools and private training structures. Public schools are present in four out of the ten regions in the country and in the District of Bamako. Private schools are present in six regions and the District of Bamako. Administration of public schools have been decentralized to the regions to improve the likelihood that students from these regions will be trained locally and will then continue to work in these areas. None of the training structures have defined strategies for attracting students. According to the managers, schools usually rely on their geographic location, students’ success rate on certification exams.

*"We do not have specific strategies*. *We do not do anything special*, *not even advertising*. *It is the performance of our graduates and the rigor in our training that attract students to our structure*.*"* (Private school manager, Bamako)

Public institutions also rely on their status as public reference schools to attract students. Entry contest dates are published and announced on radio.

*"We do not have attraction strategies*. *We are the public reference school in the region*. *Our products are well appreciated*. *Our interactions with employers and health facilities give us some reputation*, *and this attracts us students*.*”* (Head of public school in the region)

Currently, only women are attracted to and accepted in the training for midwives and obstetric nurses even though there are no rules that prevent men from registering.

#### Students’ selection procedure

Two main procedures allow training applicants to access studies: the recruitment competition and the examination of files. The competition is the main means to access public schools and is only available for Malian students. It is open to all students who graduate from secondary schools and are aged less than 22 years. Admission tests are organized in all regions. The majority of the applications usually come from Bamako and the majority of admitted students are from Bamako as well. The students are distributed between INFSS’ structures in Bamako and in the region. Students admitted through this competitive process receive a scholarship for six semesters covering tuition fees and living expenses. In addition to this process, public schools can also recruit students that can pay tuition fees without any competition.

Admission for midwifery training is open to all candidates holding the Baccalaureate Diploma, and for obstetric nurse candidates the diploma of fundamental studies (DEF) without competition. There are no age restrictions. Some institutions test French language proficiency for obstetric nurses. According to a private school manager:

*"We carry out tests for obstetric nurses*. *They must at least be able to understand and write in French*. *For midwives*, *they are holders of the baccalaureate*. *We assume they have the required level in French*.*"* (Private school manager, Bamako)

None of the structures, private and public, have preferential selection policies based on the background of the candidates.

#### School capacities and accessibility

From the document review, it appeared that Mali currently has 115 to 120 private training schools and the public school and its annexes. Most of private school managers indicated that they are training less than the capacity of their schools in regions and in Bamako. However, the total number of graduates both in public and private schools has increased over the past few years from 259 to 340 annually for midwives and from 261 per year in 2013 to a thousand per year in 2017 for obstetric nurses. This is due essentially to the increased number of training schools.

Tuition fees, according to school officials, can be a barrier to accessibility especially for rural students. Indeed, the annual costs of the tuition vary from 320 000 in public school and to 450 000 CFA (1US$ = 500CFA) in private schools, excluding living expenses. This is a large amount of money in rural areas where more than 55% live below the poverty line. However, these managers think that this training cost may help provide better training for those who are able to pay.

#### Rural exposure and curricula content for rural practice

Currently there are two different curricula in use for midwifery training. The first, objectives-based curricula, which is used in most private schools dates back to 2003. It is being used exclusively in the private sector since 2008. The second curriculum is competency based and developed by the West African Health Organization (WHAO). It has been used by the public school since 2012 and some of the private schools since 2016. For the obstetric nurses, there is only one curriculum, which was developed in 1998. According to school managers, private and public, students are trained to work in rural areas as well as in urban areas at all levels of the health system. However, most of the training takes place in urban areas. Clinical training is done in urban primary health facilities (CSComs) for first- and second-year students and mostly in referral health centers (CSRéf) and hospitals for the final-year students.

There is a policy, for both public and private schools, of exposing students to rural practice in order to familiarize them with the conditions of practice in these environments. This rural placement currently takes place at the end of the final year as part of the final examination process. Students are exposed to clinical experience in rural communities for 45 days without formal and structured teaching or supervision by teaching staff. Students are supervised by local health care providers, generally other midwives or obstetric nurses. However, despite this mandatory policy, the mandatory internships do not always take place in rural areas. Locations for internships for the public-school students are identified each year from a prospection process, and students are randomly assigned to facilities. Health facilities that are chosen to host students are generally those that are easy to reach or those that provide accommodations for students. Unlike students in public school, students from private schools benefit from little or no support for internship in rural areas. Students from private schools pay the travel cost and other expenses for living themselves. For these reasons, students from private structures are allowed to choose their internship locations. As a result, most of the students choose to stay close to or in major cities and in CSRéf.

During the rural internship, students, both from private and public schools, do clinical activities and a community diagnostic survey to identify priority health issues in the community. The rural practice is assessed through a report produced by students. Like midwifery students, obstetric nursing students are exposed to rural practice at the end of their three-year training. They are supervised by midwives and doctors.

### Students’ perceptions on willingness to practice in rural areas

These findings are based on data from the questionnaire. In total 186 students aged 17 to 42 years participated in the survey. Participants were midwifery students (39.8%, n = 74) and obstetric nursing students (60.2%, n = 112). Table 2 shows that 57% were married, more than one third were born in a rural area, 11.3% are currently living in a rural area, and 3.8% are being trained in a school located in a rural area. All students in the private sector are paying tuition. In the public schools there are students in paid tuition, but the majority are scholarship holders.

**Table 2:** Students’ characteristics and likelihood to work in rural areas.

	Obstetric nursing students n (%)	Midwifery students n (%)	Likely to go Rural n (%)
**Ages**			
Means	23.9	26.9	
Standard deviation	3.4	5.7	
**Marital status**			
Single	55 (49.1)	25 (33.8)	15 (18.8)
Married	57 (50.9)	49 (66.2)	23 (21.7)
**Place of birth**			
Urban	67 (59.8)	53 (71.6)	22 (18.3)
Rural	45 (40.2)	21 (28.4)	16 (24.2)
**Current living location**			
Urban	93 (83.1)	72 (97.3)	28 (17.0)
Rural	19 (16.9)	2 (2.7)	10 (47.6)
**Primary school location**			
Urban	78 (69.6)	50 (67.6)	23 (18.0)
Rural	34 (30.4)	24 (32.4)	15 (25.9)
**Secondary school location**			
Urban	63 (80.8)	63 (85.1)	22 (17.5)
Rural	15 (19.2)	11 (14.9)	10 (38.5)
**Professional training school location**			
Urban	105 (93.7)	74 (100.0)	34 (19.0)
Rural	7 (6.3)	0 (0.0)	4 (57.1)

#### Relation between students’ background and job preference

Most of the students mentioned that entering the training was a personal choice. Qualitative data shows that the students have an altruistic vision of their profession. The main reasons for choosing the profession are *"helping others"*, *"saving lives"*, and some have been attracted by *"the prestige of the profession"*. Most of them have relatives who are health workers.

Two-thirds (66.7%) of the students would like to work immediately upon graduation. Preferred job location were urban facilities (79.6%, n = 148). Most students would like to work in the public sector (91.4%; n = 170) and as civil servants (86.5%, n = 161). There were no differences in the intention between midwives and obstetric nurses. (Cf Table 3). They all would like to work as service providers in maternal health or nutrition or as trainers.

**Table 3:** Job preferences.

	Obstetric nursing students n (%)	Midwifery students n (%)	Total n (%)
**Would like to work upon graduation**			
Yes	78 (69.6)	46 (62.2)	124 (66.7)
**Job location preference**			
Urban	98 (87.5)	50 (67.5)	148 (79.6)
Rural	9 (8.0)	22 (29.7)	31 (16.7)
No preference	5 (4.5)	2 (2.7)	7 (3.7)
**Preferred sector of employment**			
Public	99 (88.4)	71 (95.9)	170 (91.4)
Private	13 (11.6)	3 (4.1)	16 (8.6)
**Preferred type of contract**			
Civil servant	99 (88.4)	62 (83.8)	161 (86.5)
Self-employed	5 (4.5)	0 (0.0)	5 (2.7)
Others	8 (7.1)	12 (16.2)	20 (10.8)
**Preferred work facility**			
Hospital	21 (18.7)	17 (23.0)	38 (20.5)
Csréf	43 (38.4)	28 (37.8)	71 (38.2)
Cscom	42 (37.5)	20 (27.0)	62 (33.3)
Others	6 (5.4)	9 (12.2)	15 (8.0)

Qualitative data found that students prefer to serve as civil servants in the public sector because they believe that that will make care more accessible to the population, guarantee them job stability, and bring them more respect from other care providers as a civil servant status is reputedly more difficult to reach. The majority of students would prefer to work in CSRef or hospitals because of the level of equipment, the workload and the level of service: *"We lose the hand in cscom*. *There is no equipment to do the job”* (Midwifery student). Some students would prefer to avoid working in hospitals were you only have *“complicated cases”* managed by doctor *unlike CSCom and CSRéf”* (Midwifery student).

For those who did not want to work immediately upon graduation, the reasons were the intention to undertake further training like the masters degree for midwives and midwifery diploma for obstetric nurses, or to get married and have children first. Some were enrolled in other training programs and would prefer to work with those degrees.

#### Likelihood to work in rural areas

We found that less than 20% (n = 38) of students would like to go and work in a rural area. Midwifery students were more likely to go to rural areas (32.4%, n = 24) than obstetric nursing students (12.5%, n = 14) (p = 0,01). Current living location in rural areas (47.6%, n = 10) (p = 0.003) and having professional school located in rural areas (57.1%, n = 4) (p = 0.03) and having had secondary education in rural areas (38.5%) (p = 0.01) were associated with greater intention to go in rural areas. Table 2 shows that 24.2% of students who were born in a rural area and 25.9% of those who had their primary school in rural areas show preference for practice in a rural setting but the links are not statistically significant. Marital status and place of birth did not statistically link to preference for a rural posting.

The reasons for preference for rural areas, given in the interviews, are being closer to the population, autonomy, less favoritism, and the feeling of being useful, as illustrated by the following quote of an obstetric nurse student:

“*Certainly*, *there is not much work in rural areas compared to urban areas*, *but the trust that exists between rural populations and health workers makes me prefer these environments*. *There are few health workers*, *you can easily feel useful*.” (Obstetric nurse student)

Aspects that were mentioned by students to be important to attract them to rural areas were: having the equipment to do the job properly, proximity to the family or easy access to cities and good housing conditions.

## Discussion

This study aimed to identify components of the rural pipeline applied in the Malian context to attract midwives and obstetric nurses to rural practice, the students' inclination to practice in rural health care settings and the relation between the rural pipeline components in place and intention to practice in rural areas. Key findings suggest that there is effort at the level of the curricula to provide students with rural exposure. The other components of rural pipeline like promotion of health professions among rural students and ensuring rural students’ selection into programs are not in place. Students generally prefer urban locations for living and for work. The factors associated with the intention to work in rural areas are currently living in or having attended professional or secondary school in a rural area.

Benefits of getting students from rural areas in health care profession training have been documented both in high- and in low income-countries [12,13,25]. This has been acknowledged by the government in Mali, which has led to the decentralization of public schools in regions and to granting permission to private schools to train health workers in rural areas. Nevertheless, while the proportion of midwives’ and obstetric nurses’ students has increased in the past few years, the proportion of students with rural background remains low. This could be due in part to the absence of promotion strategies of healthcare professions among students from rural areas. It could also be due to the difficulty of accessibility, both financially and geographically, of training students from rural areas. The level of poverty in most of the Malian rural settings make the training financially less accessible to rural students compared to those from urban areas if they do not get a scholarship. For the geographical accessibility, most of the schools are located in urban areas and none of the schools that train midwives are situated in rural areas. Another reason may be the limited pool of possible students in rural areas where 71% of secondary school-aged children are out of school and only 17% are attending secondary school [32].

There is no preferential selection policy currently in place in any of the training schools. Private schools rely on cost recovery from tuition payment. In public schools, half of the students were paying tuition fees. Scholarship is offered to some students in public schools by the government. But this is supposed to be done on the basis of an equal chance for all and is thus not exclusive for rural students. Most of the students who benefit are from urban areas, especially Bamako. When students are required to pay tuition fees, usually in private schools, beside the academic requirements to enter the training, the sole selection criteria applied are students’ capacities to pay tuition fees.

Due to the limited pool of possible students in rural areas and because of the limited availability of health facilities and trainers in rural areas, it may be difficult to establish training school for midwives in rural areas in Mali. What is feasible is providing rural exposure to students and having a curriculum with contents oriented to rural practice. This has proven to be effective in attracting students in rural practice in other settings [17]. Currently in Mali, there is little rural practice-oriented contents in midwives’ training curricula. The duration of rural exposure during training is very limited. It occurs only after completion of the third year of study and lasts for 45 days. Rural exposure appears to be more effective when students can benefit from earlier, frequent and longer exposure to rural practice [14]. Because the exposure occurs solely at the end of the training cycle, students actually do not benefit from structured teaching and supervision in rural areas. They do not have the opportunity to discuss and to propose adjustments in training in order to prepare them to face situations they may encounter during the rural placement. Literature suggests that to be a pull factor, rural exposure ought to provide students with a positive experience of their internships through quality coaching and working conditions. Failing this, it may, on the contrary, constitute a push factor [11]. Regarding these criteria, the current quality of rural exposure is not sufficient to ensure attraction for rural practice.

### Study limitations

It should be mentioned that our research focused on students’ preferences and these may be different from the choices of graduated midwives. Thus, further studies may be necessary to explore the relation between graduated midwives and obstetric nurses’ job preference and retention in the context of the rural pipeline in Mali. Another limitation would be the fact that we used convenience sampling and thus included those who were willing to participate, which may have caused bias.

## Conclusion

Currently, of the components of the rural pipeline (advocating health professions among rural students, ensuring that more rural students are selected into programs, developing a curriculum oriented towards rural health and rural exposure during training) examined in this study, only exposure to rural practice is implemented in the training facilities in Mali for the training of midwives and obstetric nurses. However, the duration and timing of this rural practice are not conducive to providing the desired effects on the willingness of final year students to work in rural areas. In addition, current selection procedures, especially in decentralized public schools, are not effective in providing locally trained qualified personnel for rural areas with staff shortages.

## Supporting information

S1 File Guide in French. (PDF)

S2 File Guide in English. (PDF)

S3 File Questionnaire in French. (PDF)

S4 File Questionnaire in English. (PDF)
