# Supplementary material for: Rural pipeline and willingness to work in rural areas: Mixed method study on students in midwifery and obstetric nursing in Mali
Source: PLoS One. 2019 Sep 9;14(9):e0222266. doi: 10.1371/journal.pone.0222266 (PMC6733462; doi:10.1371/journal.pone.0222266)
Supplement: S1 File — (PDF) [file pone.0222266.s001.pdf]

Guide d’entretien étudiantes sages-femmes et Infirmières obstétriciennes en fin de cycle.

Questionnaire N°__________/

Nom de l’enquêteur : Date :

Nom de l’agent de saisie : Date :

INTRODUCTION

Chers Madame et Monsieur,

Nous vous sommes très reconnaissants de votre participation à cette enquête.

Je souhaiterais vous poser quelques questions sur votre travail, votre poste, votre profession et la perception que vous avez de votre environnement de travail. Voudrez bien répondre à chacune des questions en essayant d’être le plus franc et le plus précis possible en vous basant sur votre expérience.

Merci beaucoup pour votre temps et vos efforts !

Questionnaire N°___________/ (alphanumérique, ex : Bko-000)

Identification de la structure de formation

Région : ___________________

Cercle : _____________________________

Nom de la Structure : _____________________________

Nom du lieu d'implantation (ville/village/commune) : ______________

Type de structure : ____

Profession (filière) :

- ☐ Infirmière obstétricienne
- ☐ Sage-Femme

Début de l'entretien : **Fin de l'entretien :**

Observation sur l'entretien (noter ce qui s'est passé qui a pu bloquer ou faciliter l'entretien)

Données personnelles / Background

1. Quelle est votre filière de formation ?

2. Quel âge avez-vous ?

3. Quelle est votre état matrimonial actuel ?

4. Quelle est votre lieu de naissance ?

5. Quelle est la profession de votre conjoint ?

Aspirations professionnelles et développement de carrière

6. Que comptez-vous (que planifiez-vous de faire) après l'obtention de votre diplôme ?

7. Quelles sont vos aspirations professionnelles en tant que sage-femme ou infirmière obstétricienne ? (Objectifs de carrière)

8. Quelle sont vos préférences en termes de type et de lieu de travail (Public/privé ; urbain/rural ; Cscom/CSréf/hôpitaux etc) ?

9. Quelles sont vos stratégies pour trouver un emploi de votre convenance après votre formation ?
   a. *Comment vous vous informez ou comptez-vous vous informez par rapport aux postes vacants et offre d'emploi ?*
   b. *Comment comptez-vous faire pour trouver un poste de votre préférence ?*
   c. *Sur qui comptez-vous pour vous aider à pour trouver un emploi ?*
   d. *Qu'attendez-vous des écoles ?*

10. Quelles sont les difficultés que vous prévoyez et comment vous comptez les surmonter ?

11. Que pensez-vous du travail en milieu rural ?
12. Personnellement, qu'est ce qui pourrait vous incitez à aller travailler ou à rester travailler en milieu rural ? (Qu'est ce qu'il faudrait pour vous inciter à aller travailler ou à rester travailler en milieu rural ?).
