# Supplementary material for: Rural pipeline and willingness to work in rural areas: Mixed method study on students in midwifery and obstetric nursing in Mali
Source: PLoS One. 2019 Sep 9;14(9):e0222266. doi: 10.1371/journal.pone.0222266 (PMC6733462; doi:10.1371/journal.pone.0222266)
Supplement: S2 File — (PDF) [file pone.0222266.s002.pdf]

Interview with Midwives and obstetric nurses students

Background

Location of the school?

School name

Type of schools (public or private)

Student's specialty (Midwife /Obstetric nurse)

Students' age

Matrimonial status

Place of birth

Professional aspirations and career development

1. What do you plan to do after graduation?

2. What are your career aspirations as a midwife or obstetrician?

3. What are your preferences in terms of type and place of work (public / private, urban / rural, Cscom / CSref / hospitals etc)?

4. What are your strategies for finding a job of your convenience after your training?
    a. How are you informed about job vacancies and job offers?
    b. How do you intend to find a position of your preference?
    c. Who are you counting on to help you find a job?
    d. What do you expect from your school?

5. What difficulties do you anticipate and how do you plan to overcome them?

6. What do you think of working in rural areas?
7. Personally, what might make you want to go to work or stay in rural areas? (What would it take to get you to work in rural areas?).
