# Supplementary material for: Rural pipeline and willingness to work in rural areas: Mixed method study on students in midwifery and obstetric nursing in Mali
Source: PLoS One. 2019 Sep 9;14(9):e0222266. doi: 10.1371/journal.pone.0222266 (PMC6733462; doi:10.1371/journal.pone.0222266)
Supplement: S3 File — (PDF) [file pone.0222266.s003.pdf]

Questionnaire étudiantes sages-femmes et Infirmières obstétriciennes en fin de cycle.

Questionnaire N°___________/ (alphanumérique, ex : Bko-000)

INTRODUCTION

Chère Madame,
Nous vous sommes très reconnaissants de votre participation à cette enquête. L'étude vise à s'informer des conditions d'emploi des Sages-femmes et Infirmières obstétriciennes et des conditions qui affectent leur emploi.

Comment remplir ce questionnaire

- Dans les pages qui suivent, vous trouverez un certain nombre questions et d'affirmations. Veuillez répondre à chacune des questions en cochant la case correspondante.
- Les réponses sont strictement personnelles
- Essayez d'être le plus précis possible en vous basant sur votre expérience.
- Veuillez répondre à toutes les questions s'il vous plait.

Merci beaucoup pour votre temps et vos efforts !

Questionnaire N°___________/ (alphanumérique, ex : Bko-000)

Nom de l'enquêteur : Date :

Nom de l'agent de saisie : Date :

Identification de la structure de formation

Région :

Nom de la Structure :

Nom du lieu d'implantation (ville/village/commune) :

Type de structure :

- ☐ Publique
- ☐ Privée

Quelle est votre filière de formation ?

- ☐ Infirmière obstétricienne
- ☐ Sage-Femme

Données personnelles

1. Cocher la case correspondant au sexe du répondant
   - Femme.................................... ☐
   - Homme.................................... ☐

2. Quel âge avez-vous ? Ans____________(en années)

3. Quelle est votre état matrimonial actuel (*cocher la case correspondante*) ?
   - N'a jamais été marié(e)............. ☐
   - Marié(e)..................................... ☐
   - Divorcé(e) .... ☐
   - Veuf/veuve .... ☐

4. Quelle est votre lieu de naissance *(cocher la réponse qui s'applique à votre cas)* ?
   - ☐ Bamako
   - ☐ Chef-lieu de région (capitales régionales)
   - ☐ Rural (cercle, commune, village)
   - ☐ En dehors du Mali

5. Où avez-vous fait vos études primaires *(cocher la réponse qui s'applique a votre cas)* ?
   - ☐ Bamako
   - ☐ Chef-lieu de région (capitales régionales)
   - ☐ Rural (autre que chef-lieu de région)
   - ☐ En dehors du Mali

6. Où avez-vous fait vos études secondaires (uniquement pour les sages-femmes) ?
   - ☐ Bamako
   - ☐ Chef-lieu de région (capitales régionales)
   - ☐ Rural (autre que chef-lieu de région)
   - ☐ En dehors du Mali

7. Quel est votre lieu de résidence habituellement ?
   - ☐ Bamako
   - ☐ Chef-lieu de région (capitales régionales)
   - ☐ Rural (autre que chef-lieu de région)
   - ☐ En dehors du Mali

8. Quelle est la profession de votre conjoint ? (*Seulement pour les personnes mariées)*
   ____

Aspirations professionnelles

A présent j'aimerais vous poser quelques questions relatives à vos aspirations professionnelles générales.

Motivation pour le choix de la profession

9. Pourquoi avez-vous choisi de devenir sages-femmes/infirmière obstétricienne *(cocher les trois réponses qui se rapportent le plus à votre cas)* ?

- ☐ Amour de la profession
- ☐ Encouragement des autres
- ☐ La garantie d'avoir du travail
- ☐ Salaire élevé
- ☐ Bonnes possibilités de promotion
- ☐ Un travail où vous pouvez être indépendant
- ☐ Un travail où vous pouvez aider les autres
- ☐ Un emploi avec temps de travail flexible
- ☐ Un emploi avec beaucoup de temps libre
- ☐ Un emploi avec contact humain
- ☐ Possibilités de travail à temps partiel
- ☐ Un travail où vous pouvez créer et former
- ☐ Travail en équipe.
- ☐ Autres : ______________________

10. Qu'est ce qui a influencé votre choix (à devenir sage-femme/infirmière obstétricienne *(cocher la réponse qui se rapporte à votre cas) ?*

- ☐ Parents (père, mère ; sœurs, frères)
- ☐ Conjoint (mari…)
- ☐ Professionnelles de santé
- ☐ Amis
- ☐ Autres étudiantes
- ☐ Personne
- ☐ Autres (à préciser) ________________________________

Intention de travailler dans le domaine de la santé ou autre secteur

11. Avez-vous l'intention, à l'obtention de votre diplôme, de chercher du travail tout de suite ?
    - ☐ Oui (si oui aller à la question 13)
    - ☐ Non (si oui aller à la question 12)

12. Qu'aimeriez-vous faire (qu'avez-vous l'intention de faire) tout de suite après l'obtention de votre diplôme *(cocher la réponse qui se rapporte à votre cas)* ?
    - ☐ Autres études
    - ☐ Voyager
    - ☐ Se marier
    - ☐ Avoir des enfants
    - ☐ Autres (préciser)________________________

13. Avez-vous l'intention de travailler dans le domaine de la santé ?
    - ☐ Oui (aller à la question 15)
    - ☐ Non

14. Si non, dans quel domaine aimeriez-vous (avez-vous l'intention) de travailler ?

    ____

15. Où aimeriez-vous travailler (*cocher la réponse qui se rapporte à votre cas*) ?
    - ☐ Bamako
    - ☐ Hors Bamako

☐ Autre (à préciser) ____

16. Dans quel secteur d'emploi préfèreriez-vous travailler *?*

☐ Public
☐ Privé

17. Quel type de statut préfèreriez vous *?*

☐ Fonctionnaire (état ou collectivité)
☐ Contractuel (ONG ou autre)
☐ Fonds Asaco
☐ Travaillez à votre propre compte
☐ Autres (à préciser) ……………………………………………

18. Quel type d'emploi aimeriez-vous avoir ?

☐ Soins
☐ Autres (à préciser) _________________________

19. Dans quel type de structure/niveau de structure aimeriez-vous travaillez ?

☐ Cscom
☐ CSRéf
☐ Hôpitaux
☐ Clinique (privées)
☐ Confessionnelle
☐ Autres (à préciser) _________________________

20. Quelles stratégies comptez-vous utiliser pour avoir du travail dans le domaine et le secteur que vous voulez ?

………

………

………

………

………

Attentes en termes de développement de carrière

21. Combien de temps pensez-vous travailler comme sage-femme/Infirmière obstétricienne ?

☐ Moins de cinq ans
☐ Plus de cinq ans
☐ Je ne sais pas

22. Avez-vous l'intention d'entreprendre d'autres études?

☐ Oui
☐ Non

23. Si oui dans quel domaine avez-vous l'intention d'entreprendre ces études.

☐ Dans le domaine de la santé
☐ Autres domaine (préciser)_____________________________
