# Supplementary material for: Rural pipeline and willingness to work in rural areas: Mixed method study on students in midwifery and obstetric nursing in Mali
Source: PLoS One. 2019 Sep 9;14(9):e0222266. doi: 10.1371/journal.pone.0222266 (PMC6733462; doi:10.1371/journal.pone.0222266)
Supplement: S4 File — (PDF) [file pone.0222266.s004.pdf]

Questionnaire: midwives and obstetric nurses students

Questionnaire N°___________/

Background

Location of the school:
School name:
Type of schools:

- ☐ Public
- ☐ Private

Specialty

- ☐ Midwife
- ☐ Obstetric nurse

How old are you (in years)?

1. What is your current marital status (check the corresponding box)?

Never Married .... ☐
Married.................................. ☐
Separted................................. ☐
Widowed .... ☐

2. What is your place of birth (check the answer that applies to you)?
   - ☐ Bamako
   - ☐ Urban area in region
   - ☐ Rural areas
   - ☐ Outside Mali

3. Where did you go to primary school (check the answer that applies to you)?
   - ☐ Bamako
   - ☐ Urban area in region
   - ☐ Rural areas
   - ☐ Outside Mali

4. Where did you go to high school (midwives only)?
   - ☐ Bamako
   - ☐ Urban area in region
   - ☐ Rural areas
   - ☐ Outside Mali

5. What is your usual place of residence?
   - ☐ Bamako
   - ☐ Urban area in region
   - ☐ Rural areas
   - ☐ Outside Mali
6. What is your spouse's occupation? (Only for married people)

Motivation for choosing the profession

7. Why did you choose to become a midwife / obstetric nurse (check the three answers that are most relevant to your case)?

☐Love of the profession

☐ Encouragement of others

☐ The guarantee of having a post

☐ High salary

- Good opportunities for promotion
- A job where you can be independent
- A job where you can help others
- A job with flexible working hours
- A job with a lot of free time
- A job with human contact
- Possibilities for part-time work
- A job where you can create and train
- Team work.

8. What influenced your choice (to become a midwife / obstetrician (tick off the answer that pertains to your case)?

- Parents (father, mother, sisters, brothers)
- Spouse (husband ...)
- Health professionals
- Friends
- Other students
- Person
- Other (to be specified) ____________________

Intention to work in health sector

9. Do you intend, upon graduation, to look for work right away?
   - Yes (if yes go to question 11)
   - No (if yes go to question 10)

10. What would you like to do (what do you intend to do) right after graduation (check the answer that pertains to your case)?
    - Other studies
    - Traveling
    - Getting married
    - Have children
    - Other (specify) ____________________

11. Do you intend to work in the field of health?
    - Yes (go to question 15)
    - No

12. If no, in what area would you like (do you intend) to work?

13. Where would you like to work (check the answer that pertains to your case)?
    - Bamako
    - Urban areas in region
    - Rural areas
    - Other (to be specified)

14. In which sector of employment would you prefer to work?
    - Public
    - Private

15. What kind of status would you prefer?
    - civil servant
    - Contractual (NGO or other)
    - Asaco Fund
    - Self employed
    - Other (to be specified) ....

16. What kind of job would you like to have?
    - Care

□ Other (to be specified) ______________________________

17. In what type of structure / level of structure would you like to work?
    - □ Cscom
    - □ CSRef
    - □ Hospitals
    - □ Clinic (private)
    - □ Confessional
    - □ Other (to be specified) ______________________________

18. What strategies do you plan to use to get work in the field and sector you want?

Expectations in terms of career development

19. How long do you think of working as a midwife / obstetric nurse?
    - □ Less than five years
    - □ More than five years
    - □ I do not know

20. Do you intend to undertake further studies?
    - □ Yes
    - □ No
        a. If yes, in which area do you intend to undertake these studies?
    - □ In the field of health
    - □ Other area (specify)
